# Psychometric validation of ERA-12 and aging expectations among PLHIV in Kazakhstan

**DOI:** 10.3389/fpubh.2026.1733639

**Published:** 2026-03-19

**Authors:** Balnur Iskakova, Deborah Gustafson, Aigerim Alimbekova, Anarkhan Nurkerimova, Gulmira Kalzhanbayeva, Gulnara Nugumanova, Ademi Sarsembiyeva, Jack DeHovitz, Recai Yucel, Zhamilya Nugmanova

**Affiliations:** 1 Department of Health Policy and Management, School of Public Health, Asfendiyarov Kazakh National Medical University, Almaty, Kazakhstan; 2 Department of Neurology, SUNY Downstate Health Sciences University, New York, NY, United States; 3 Almaty Center for AIDS Prevention and Control (ACAPC), Almaty, Kazakhstan; 4 Department of Epidemiology, School of Public Health, Asfendiyarov Kazakh National Medical University, Almaty, Kazakhstan; 5 Department of Medicine, SUNY Downstate Health Sciences University, New York, NY, United States; 6 Department of Epidemiology and Biostatistics, Temple University, Barnett College of Public Health, Philadelphia, PA, United States

**Keywords:** aging expectations, Central Asia, HIV, Kazakhstan, psychometrics, stigma

## Abstract

**Background:**

With longer life expectancy due to antiretroviral therapy, people living with HIV (PLHIV) face age-related conditions and psychosocial challenges that influence their perceptions of aging. Yet, no study has examined aging expectations in Kazakhstan or the broader Central Asian region, particularly among PLHIV. This study aimed to assess the psychometric properties of the Russian version of the 12-item Expectations Regarding Aging (R-ERA-12) scale, a brief, internationally validated multidimensional instrument, and to examine aging expectations among PLHIV in Almaty.

**Methods:**

Data were collected from 100 PLHIV aged 40 years and older enrolled in a follow-up pilot study at the Almaty Centre for AIDS Prevention and Control between 2023 and 2024. Sociodemographic information, R-ERA-12 responses, stigma, quality of life measures, and HIV-related laboratory data were collected. Confirmatory factor analysis (CFA) tested the three-factor structure representing physical, mental, and cognitive expectations. Model fit was evaluated using standard indices, and reliability and validity were examined through internal consistency and factor-based indicators. Linear regression models were applied to identify factors associated with total R-ERA-12 scores.

**Results:**

CFA confirmed that the three-factor model provided a good fit, and the scale demonstrated high internal consistency (*α* = 0.93). Subscale reliability was also strong (α = 0.84–0.87), with the average variance explained by each factor ranging from 57 to 64%, indicating good construct validity. Participants reported generally low expectations regarding aging, particularly in the physical health domain. In multivariable analysis, higher levels of internalized HIV stigma were significantly associated with more negative aging expectations (*β* = −1.91, *p* = 0.03).

**Conclusion:**

The R-ERA-12 demonstrated robust psychometric properties and is a reliable instrument for assessing aging expectations among PLHIV. Findings highlight notably low aging expectations and suggest that addressing internalized stigma could help foster more positive beliefs about aging, supporting the integration of ERA-12 into clinical and psychosocial programmes for PLHIV in Kazakhstan and similar settings.

## Introduction

People with HIV (PLHIV) are experiencing increased longevity due to significant advancements in HIV treatment and care. These advancements have shifted the focus from managing HIV as a terminal illness to addressing other health conditions associated with aging and their interaction with HIV. Previous studies indicate that PLHIV experience an earlier risk of age-related conditions compared to the general population, including frailty, cardiovascular and metabolic disorders, cancers, neurodegenerative diseases, and bone-related conditions (1, 2). Even among individuals with sustained viral suppression through adherence to antiretroviral therapy (ART), disparities in comorbidity-free life expectancy persist relative to the general population (2). This excess burden of age-related conditions reflect a pattern of accelerated or premature aging among PLHIV, influenced by the virus itself, ART-related toxicity, co-infections, and behavioral, lifestyle, and/or psychosocial factors (2).

Although aging is commonly associated with declines in physical and functional health, geriatricians and gerontologists are increasingly focusing on positive constructs, such as healthy aging and intrinsic capacity. According to the World Health Organization (WHO), intrinsic capacity refers to “the composite of all the physical and mental capacities that an individual can draw on,” serving as the foundation for healthy aging, which is defined as “the process of developing and maintaining the functional ability that enables well-being in older age” (3).

Health outcomes in later life are influenced not only by objective health measures but also by subjective perceptions of health and aging. Beliefs and expectations about aging have been shown to significantly affect both physical and psychological outcomes. Individuals with more positive expectations regarding aging tend to experience better overall health, enhanced well-being, and even increased longevity (4, 5). Positive aging expectations are also linked to improved social outcomes, such as greater engagement in community life and reduced feelings of loneliness (6). Conversely, internalized ageist stereotypes, particularly those suggesting inevitable physical decline, may negatively affect self-perception and functioning in later life. As theorized by Levy and colleagues, these stereotypes, if adopted early, can manifest as self-fulfilling prophecies, leading to diminished physical and social performance in older age (7). In addition to age-related stereotypes, PLHIV may experience HIV-related stigma, which can further shape perceptions of health and aging. Internalized stigma and experiences of discrimination in healthcare settings have been associated with poorer mental health, reduced self-esteem, and lower quality of life among PLHIV (8, 9). Such psychosocial stressors may negatively impact how individuals perceive their future health and aging trajectory, potentially contributing to less positive expectations regarding aging.

In Kazakhstan, research on aging, particularly among marginalized groups such as PLHIV, remains limited. Existing studies have largely focused on specific health outcomes and aspects of quality of life among older adults, including cognitive functioning in community-dwelling seniors, readiness for physical activity, and bone health among the older adults (10–13). Recent governmental and community initiatives, such as Active Longevity Centers, aim to promote healthy aging through free health, educational, and social activities (14). However, participation in such programs assumes baseline positive aging expectations, which may be lacking among individuals facing chronic illnesses such as HIV, social stigma, or socioeconomic vulnerabilities. Studying aging expectations among PLHIV is important not only to understand the unique challenges faced by this population but also to identify disparities relative to the general public. This knowledge can inform targeted interventions to promote healthy aging and improve quality of life, ensuring that PLHIV can achieve aging outcomes comparable to the broader population.

Commonly used to assess aging expectations are the 38-item “Expectations Regarding Aging” instrument (ERA-38) and its shorter version, the ERA-12 (15). These instruments were developed and validated primarily in Western populations, and more recently in Japan, Iran, and China (16–18). These studies largely focused on older adults, with only a few studies involving middle-aged and younger individuals. To our knowledge, no study has evaluated the psychometric properties of the ERA-12 in Russian (R-ERA-12). Therefore, the present study aims to: (1) evaluate the psychometric properties of the R-ERA-12 scale, and (2) assess aging expectations among PLHIV in Almaty, Kazakhstan, along with factors associated with R-ERA-12 scores. By validating the R-ERA-12 and examining aging expectations in this context, the study addresses an important regional gap and contributes evidence from an underrepresented setting to the global literature on aging with HIV. The validated instrument may also facilitate future research in other Russian-speaking settings, including countries in Central Asia.

## Methods

### Study design and setting

Two consecutive pilot studies were conducted between 2022 and 2024 at the Almaty Centre for AIDS Prevention and Control (ACAPC), the primary HIV care provider for the Almaty region, serving both urban and rural populations.

The first pilot study (2022–2023) was a cross-sectional investigation of cardiovascular health among 150 PLHIV aged ≥40 years receiving routine care at ACAPC (19). Participants were consecutively recruited during routine clinic visits over approximately 6 months in 2023. Data collected included self-reported sociodemographic characteristics, health outcomes, clinical and HIV-related factors, mental and cognitive health indicators, quality of life measures, and fasting blood samples. Participants were asked whether they consented to re-contact for future related studies.

The second pilot study (2023–2024), providing the basis for the present analysis, was a follow-up of the initial cohort. Of those who consented to re-contact, 100 participants were successfully re-enrolled; nearly all eligible invited participants agreed to participate.

### Data collection

Data were collected through standardized, physician-administered questionnaires and blood tests for HIV-related biomarkers at ACAPC. Study staff underwent structured training to ensure consistent administration of all instruments. All surveys were administered in Russian, the predominant language of communication among adults aged ≥40 years in Kazakhstan, particularly in urban settings such as Almaty. Data were recorded and managed using the Centers for Disease Control and Prevention’s KoBo Toolbox platform.^1^

### Measures

#### Outcome variable

Expectations about aging were measured using the 12-item Russian version of the ERA-12 (R-ERA-12), which assesses perceptions of aging across three domains:

- Physical health
- Mental health
- Cognitive function

The English version was translated into Russian and back-translated to English by bilingual investigators (BI, ZN) to ensure semantic equivalence. Each item was rated on a four-point scale from “Definitely true” to “Definitely false.” Subscale scores (physical, mental, and cognitive) were computed based on four items each. The total R-ERA-12 score was derived from all 12 items and transformed to a 0–100 scale, with higher scores reflecting more positive expectations regarding aging (15).

### Covariates

Sociodemographic variables included:

- Age and sex
- Education, categorized as:
- Secondary education (incomplete or complete secondary school)
- Vocational/specialized secondary education (vocational/technical college or incomplete higher education)
- Higher/postgraduate education (completed undergraduate or graduate degrees)
- Employment status: employed (full- or part-time), unemployed, or other (retired, disabled, or other)
- Housing stability: stable (own home or living with relatives) versus unstable/vulnerable (shelter, assisted housing, or street living)

HIV-related clinical indicators:

HIV-related indicators were assessed in line with international and national thresholds (20):

- CD4 cell count (>500, 200–500, <200 cells/mm^3^)
- Viral load (≤1,000 or >1,000 copies/mL)
- Duration of HIV infection (years since diagnosis)

Multimorbidity was defined as the presence of ≥3 self-reported chronic conditions (excluding HIV), such as:

- Stroke
- Heart disease
- Chronic lung disease
- Diabetes
- Hypertension
- Hypercholesterolemia
- Thyroid or liver disease
- Traumatic brain injury
- Bone disease

Quality of life was assessed using the WHO Quality of Life Scale (WHOQOL-26) (21). While clinical thresholds were determined according to established cut-offs, the analysis focused on the overall score. One WHOQOL-26 item related to financial strain (“Do you have enough money to meet your needs?”) was analyzed separately in regression models.

HIV Stigma included:

- Internalized HIV stigma: measured using a six-item subscale from the Stigma Index 2.0 (e.g., “My HIV infection makes me feel dirty”) (22). Responses (“agree”/“disagree”) were summed (0–6), with higher scores indicating greater stigma.
- Clinic-based stigma: measured using a seven-item scale assessing negative experiences in healthcare settings (e.g., “You were denied medical services due to your HIV status”), with higher scores indicating greater perceived stigma.

### Statistical analysis

Descriptive statistics were summarized as frequencies and percentages for categorical variables and means ± standard deviations for continuous variables. Group differences by sex were examined using chi-square tests (*χ*^2^) for categorical variables and independent-sample t-tests for continuous variables.

A confirmatory factor analysis (CFA) using robust maximum likelihood estimation (MLR) validated the three-factor structure of the R-ERA-12 (physical, mental, cognitive). The model was specified *a priori* based on the original ERA-12 structure (11–14). Factor covariances were freely estimated. Given the pilot nature and modest sample size, no post-hoc modifications or correlated residuals were specified. Model fit was evaluated using the chi-square (*χ*^2^) test, Comparative Fit Index (CFI), Tucker–Lewis Index (TLI), Root Mean Square Error of Approximation (RMSEA), and Standardized Root Mean Square Residual (SRMR), with acceptable fit defined as CFI/TLI ≥ 0.90, RMSEA ≤0.08, and SRMR ≤0.08. Reliability was assessed using Cronbach’s alpha. Convergent validity was assessed via standardized factor loadings, AVE ≥ 0.50, and CR ≥ 0.70; discriminant validity was examined by comparing the square roots of AVEs with inter-factor correlations.

To identify determinants of aging expectations, univariate linear regression was first conducted with the R-ERA-12 total score and all covariates (sociodemographics, HIV indicators, stigma). Variables with *p* < 0.20 were included in a multivariate model, where *p* < 0.05 defined significance. Age and sex were retained in all models regardless of significance. Analyses were conducted using R 4.4.1 (R Core Team, 2024).

### Ethical considerations

Ethical approval for this study was obtained from the Kazakh National Medical University Ethics Committee (IRB session No. 8/144) prior to data collection. Written informed consent was obtained from all participants in accordance with the Declaration of Helsinki.

## Results

### Sample characteristics

The study sample consisted of a greater proportion of males (58%), with a mean age of 50 years (SD = 7.87). As summarized in Table 1, nearly half of the respondents (41%) had an adequate immune status (CD4 > 500 cells/mm^3^), while 16% exhibited advanced immunodeficiency (CD4 < 200 cells/mm^3^). Most individuals (88%) demonstrated effective viral suppression, defined according to national standards as ≤1,000 copies/mL (20). Multimorbidity was observed in 38% of participants. No significant sex-based variations were identified across descriptive characteristics.

**Table 1:** Characteristics of study participants by sex, including sociodemographic, behavioral, and HIV-related clinical variables (*N* = 100).

Variables	*N* (%)	Female	Male	*p*-value
		*N* = 42	*N* = 58	
Age category (years)				
40–49	56 (56%)	28 (28%)	28 (28%)	0.17
50–59	31 (31%)	11 (11%)	20 (20%)	
60–69	9 (9%)	3 (3%)	6 (6%)	
≥70	4 (4%)	0 (0%)	4 (4%)	
Education^a^				
High and postgraduate	25 (25%)	10 (10%)	15 (15%)	0.39
Specialized secondary	16 (16%)	9 (9%)	7 (7%)	
Secondary	59 (59%)	23 (23%)	36 (36%)	
Employment				
Employed	39 (39%)	18 (18%)	21 (21%)	0.28
Other (retired, disabled, and other)	22 (22%)	6 (6%)	16 (16%)	
Unemployed	39 (39%)	18 (18%)	21 (21%)	
Living arrangement^b^				
Stable housing	88 (88%)	36 (36%)	52 (52%)	0.77
Unstable/vulnerable housing	12 (12%)	6 (6%)	6 (6%)	
Smoking				
Not at all	14 (14%)	8 (8%)	6 (6%)	0.18
Some days	21 (21%)	11 (11%)	10 (10%)	
Everyday	65 (65%)	23 (23%)	42 (42%)	
CD4 cell count (cells/mL)				
>500	41 (41%)	17 (17%)	24 (24%)	0.98
500–200	43 (43%)	18 (18%)	25 (25%)	
<200	16 (16%)	7 (7%)	9 (9%)	
HIV Viral Load (copies/mL)				
≤1,000	88 (88%)	39 (39%)	49 (49%)	0.36
>1,000	11 (11%)	3 (3%)	8 (8%)	

a High or postgraduate education-completed undergraduate or graduate degrees; Specialized secondary education vocational or technical college, or incomplete higher education; Secondary education-incomplete and complete secondary school.

b Stable-own home or with relatives; Unstable/vulnerable housing-shelter, assisted housing, street.

### Objective 1: psychometric properties of the R-ERA-12

#### Confirmatory factor analysis

The CFA results demonstrated that the hypothesized three-factor structure of the R-ERA-12 provided a good fit to the data without post-hoc model modification. (*χ*^2^ = 56.67, *p* = 0.27; CFI = 0.985; TLI = 0.980; RMSEA = 0.045 [90% CI: 0.00–0.10]; SRMR = 0.057). All 12 items loaded significantly and strongly on their respective latent constructs, confirming the adequacy of the three-factor model (Table 2). Convergent validity was supported by standardized loadings >0.50 and acceptable AVE and CR values. Discriminant validity was confirmed as the square roots of the AVEs exceeded inter-factor correlations. Internal consistency was high for both the total scale and subscales (*α* = 0.84–0.93).

**Table 2:** Confirmatory factor analysis of the R-ERA-12 (*N* = 100).

No.	Factor/Item	Standardized loading
1.	Physical health	
	(*α* = 0.84; CR = 0.84; AVE = 0.53)^a^	
	1. When people get older, they need to lower their expectations of how healthy they can be.	0.689
	2. The human body is like a car: when it gets old, it gets worn out.	0.743
	3. Having more aches and pains is an accepted part of aging.	0.881
	4. Every year that people age, their energy levels go down a little more.^a^	0.553
2.	Mental health	
	(α = 0.86; CR = 0.87; AVE = 0.63)^a^	
	5. I expect that as I get older I will spend less time with friends and family.	0.802
	6. Being lonely is just something that happens when people get old.	0.737
	7. Quality of life declines as people age.	0.795
	8. It’s normal to be depressed when you are old.	0.796
3	Cognitive health	
	(α = 0.87; CR = 0.88; AVE = 0.64)^a^	
	9. I expect that as I get older I will become more forgetful.	0.772
	10. It’s an accepted part of aging to have trouble remembering names.	0.807
	11. Forgetfulness is a natural occurrence just from growing old.	0.87
	12. It is impossible to escape the mental slowness that happens with aging.	0.723

^a^α, Cronbach’s alpha; CR, Composite reliability; AVE, Average variance extracted.

**Figure 1:**
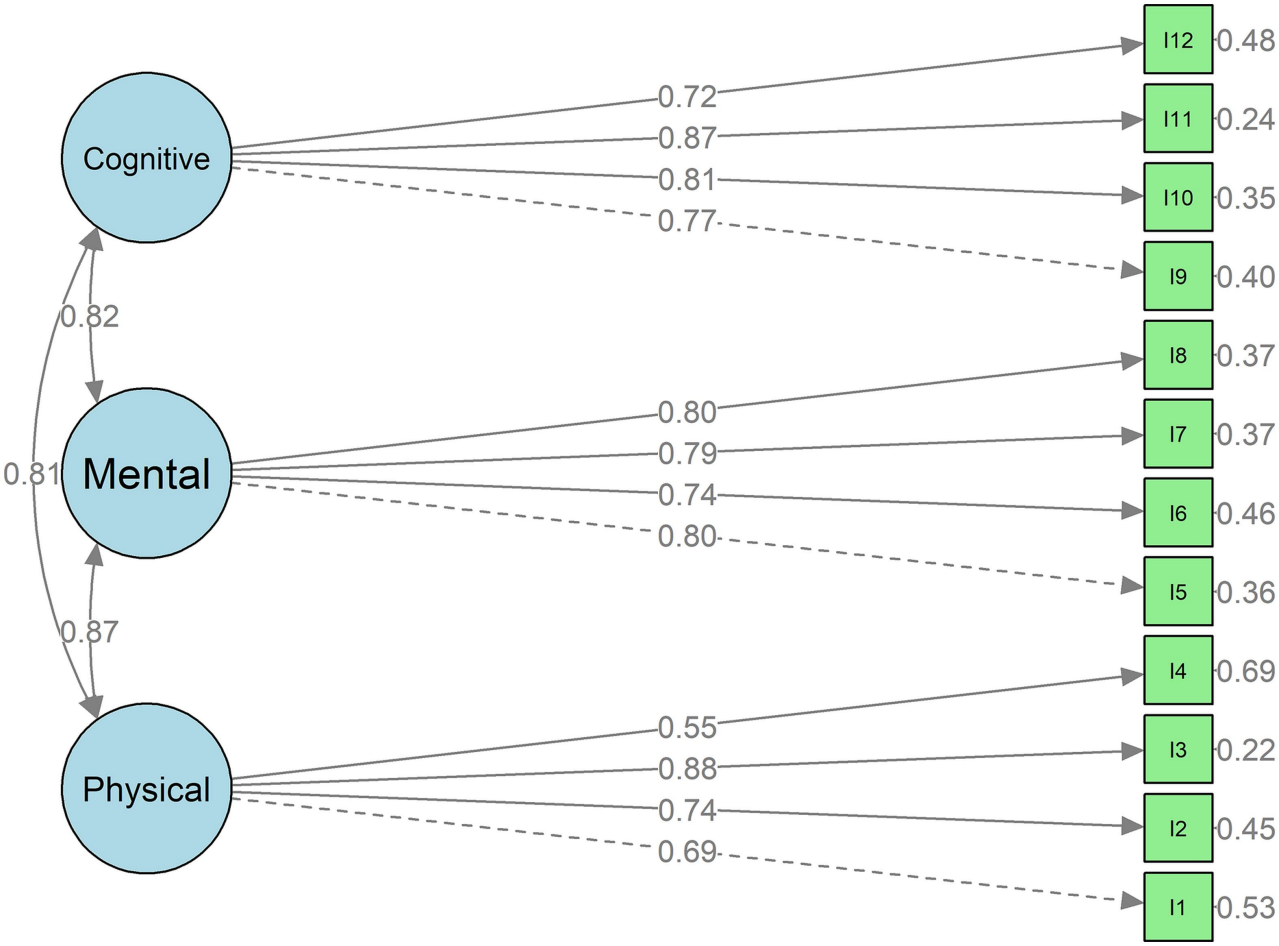
Path diagram of the three-factor model of the R-ERA-12 scale items (I1–I12).

As shown in Figure 1, the three latent factors were moderately to strongly interrelated (*r* = 0.81–0.87), reflecting conceptual overlap while maintaining distinct dimensions. Internal consistency was excellent for the total scale (*α* = 0.93) and subscales (Physical = 0.84, Mental = 0.86, Cognitive = 0.87), confirming the strong reliability of the R-ERA-12 in this population.

### Objective 2: aging expectations and associated factors

#### Descriptive analysis of aging expectations

Overall, the mean total R-ERA-12 score was 29.12 (SD = 18.34), indicating generally low expectations regarding aging. Subscale mean scores were: mental health 31.25 (SD = 22.47), physical health 27.49 (SD = 20.94), and cognitive health 26.18 (SD = 19.70).

**Figure 2:**
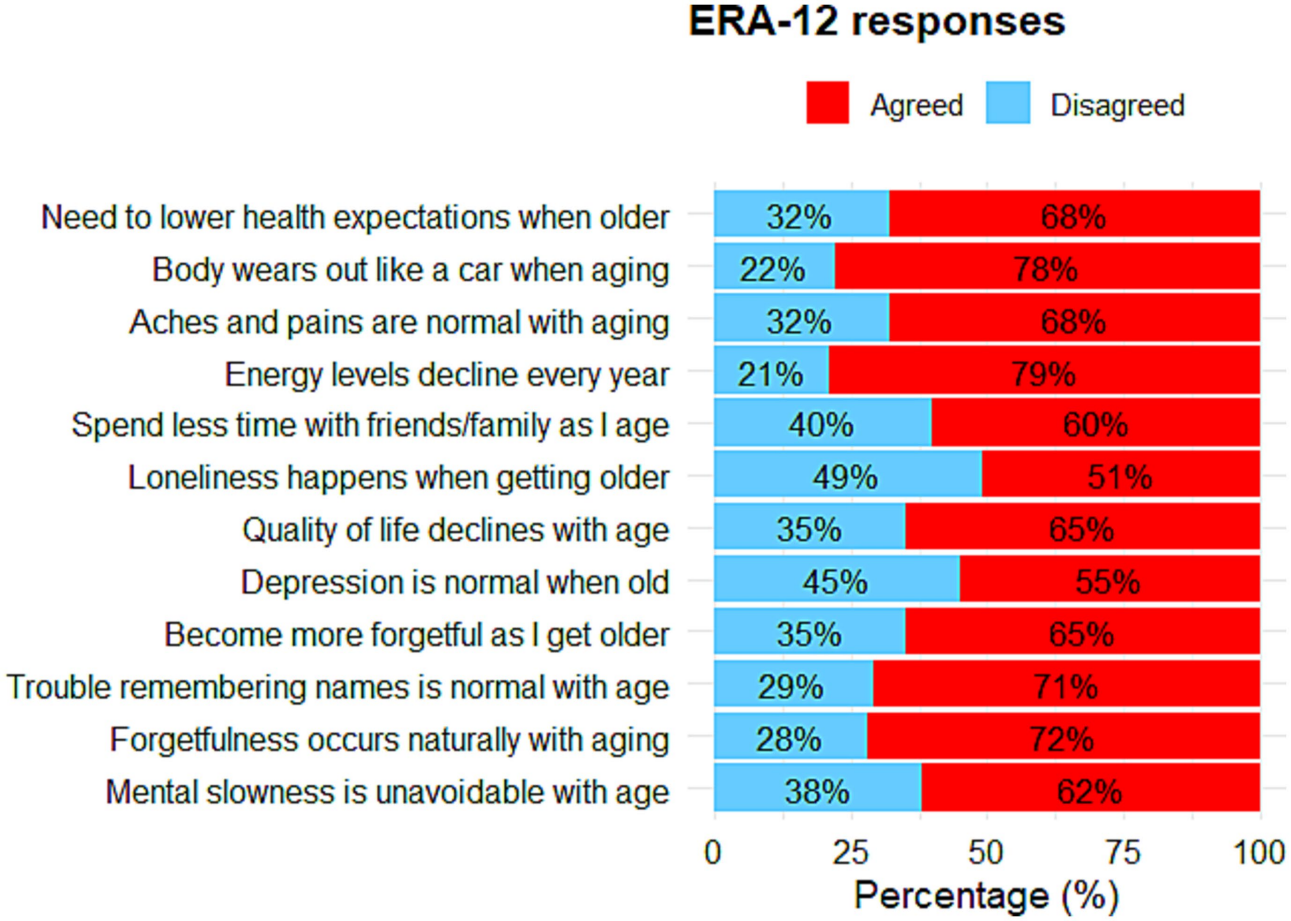
Percentage distribution of responses to individual items of the R-ERA-12.

As shown in Figure 2, response patterns were predominantly towards agreement with the given negative statements. Notably, a large proportion of respondents held negative expectations regarding their physical health, as 79% agree that energy levels decline with age, and similarly, 78% believe that the body wears out over time.

### Factors associated with aging expectations

Education, employment, and internalized HIV stigma were identified as significant (*p* < 0.20) predictors of R-ERA-12 score in univariate linear regression models and were therefore included in the multiple linear regression model adjusted for age and sex. The results of the final model are presented in Table 3. Consistent with the univariate findings, only internalized HIV stigma remained significantly associated with the R-ERA-12 total score in the multivariable model (*β* = −1.91, 95% CI −3.63 to −0.19, *p* = 0.03). Specifically, higher levels of internalized stigma were associated with lower expectations regarding aging, indicating that participants with less stigma reported more positive expectations of aging.

**Table 3:** Results of multiple linear regression analyses predicting R-ERA-12 total score from sociodemographic and psychosocial variables (*N* = 100).

Covariates	Estimate (*β*)	SE	*t*	*p*	95% CI
Age	−0.29	0.28	−1.03	0.30	−0.84, 0.27
Sex					
Male vs. Female	0.95	3.98	0.24	0.81	−6.96, 8.87
Internalised HIV stigma	−1.91	0.86	−2.21	0.03	−3.63, −0.19
Overall quality of life	0.14	0.12	1.22	0.22	−0.09, 0.37
Education^a^					
Specialized secondary vs. Secondary	−0.09	5.84	−0.02	0.98	−11.70, 11.52
High and postgraduate vs. Secondary	4.22	4.91	0.86	0.39	−5.56, 14.00
Employment^b^					
Employed vs. Unemployed	6.2	5.91	1.05	0.29	−5.56, 17.95
Other vs. Unemployed	3.69	4.58	0.81	0.42	−5.42, 12.79

a High or postgraduate education-completed undergraduate or graduate degrees; Specialized secondary education -vocational or technical college, or incomplete higher education; Secondary education-incomplete and complete secondary school.

b Other- retired, disabled, and other.

## Discussion

This study aimed to evaluate the psychometric properties of the 12-Item R-ERA-12 and to examine aging expectations among PLHIV in Kazakhstan. Our findings demonstrate that the R-ERA-12 is a reliable and valid instrument for use in this population, confirming strong psychometric properties that align with previous international studies. Moreover, PLHIV in our sample reported alarmingly low expectations regarding aging, particularly in the domain of physical health, consistent with findings from other regions.

The original ERA-12 study by Sarkisian et al. (15) reported Cronbach’s alpha coefficients exceeding 0.75 for each subscale and an overall *α* of 0.88. In the present study, internal consistency reliability for the total score and subscales ranged between 0.84 and 0.93. These values are comparable to or slightly higher than those reported in previous validations of the ERA-12 in English, Persian, Chinese, and Japanese populations (0.70–0.89) (16–18). Factor loadings in our study ranged from 0.55 to 0.88, marginally lower than those in the original study and closely aligned with those found in the Japanese and Persian versions (16, 17). These results collectively confirm that the R-ERA-12 retains robust psychometric properties, even among individuals with chronic health conditions such as HIV, and is suitable for use in research contexts.

Descriptive analyses indicated generally low aging expectations across all domains, with somewhat lower scores in physical health, consistent with prior research among community-dwelling adults and older PLHIV in high-income countries (15, 23–26). Among the factors examined, internalized HIV stigma was the only variable that remained significantly associated with poorer aging expectations in the multivariable model. Although prior research suggests that multimorbidity, depressive symptoms, financial hardship, and lower quality of life are linked to more negative aging perceptions among PLHIV, our findings identified internalized stigma as the primary correlate (27–29). There are several possible explanations. First, our sample is relatively younger, and many participants appear to be in a stable clinical condition, which may limit variation in health status and thus reduce the statistical power to detect effects of comorbidity. Second, in younger or healthier PLHIV, internalized stigma may exert a more immediate psychological impact on perceptions of aging, whereas the downstream effects of physical decline, functional impairment, or economic hardship may accumulate more slowly and become evident only in older or more clinically compromised groups. For example, a study comparing older and younger PLHIV reported that older participants experienced less internalized stigma than those younger than 40 years, possibly because older individuals had learned to cope with and manage the negative aspects of living with HIV and stigma over time (30).

Internalized HIV stigma remains a persistent challenge that continues to undermine both HIV care and broader health outcomes. Individuals may internalize negative societal attitudes and beliefs about their condition, leading to feelings of shame, guilt, self-blame, social withdrawal, and reduced engagement with healthcare services (31, 32). According to Earnshaw and Chaudoir (33), these psychological processes are key mechanisms through which stigma influences health, as internalized stigma can alter self-concept, reduce self-efficacy, and shape expectations for the future (33). Furthermore, PLHIV often experience multiple and intersecting forms of stigma. For instance, our earlier study conducted among healthcare workers in Kazakhstan suggested that stigma was more closely related to moral judgments of key affected populations (e.g., sex workers, men who have sex with men) than to HIV itself (34). In addition, older long-term survivors of HIV have reported experiencing stigmatizing attitudes from others due to perceptions about their lifestyle at an older age (35). These overlapping stigmas can create a compounded burden, influencing how PLHIV perceive their aging trajectory and reinforcing negative expectations regarding physical decline, cognitive impairment, and social isolation. Clinicians should be aware that older PLHIV may experience stigma related both to HIV and to aging, which may influence engagement with care and perceptions of future health. At the public health level, interventions promoting healthy aging among PLHIV should integrate stigma-reduction approaches that explicitly address both HIV and aging-related stereotypes.

To our knowledge, this is the first study to evaluate the psychometric properties of the ERA-12 scale in Russian and to explore aging expectations among PLHIV in the Central Asian region, including Kazakhstan. Cross-cultural validation of psychometric instruments requires careful consideration of linguistic and generational context. In Kazakhstan, language use varies by age group. Many older adults were educated primarily in Russian during the Soviet period, and Russian continues to be widely used in healthcare and research settings, particularly in large urban centres. Concurrently, the growing prominence of the Kazakh language among younger generations reflects ongoing sociolinguistic shifts. Validating the ERA-12 in Russian therefore addresses the linguistic realities of today’s middle-aged and older PLHIV while supporting the development of culturally and contextually appropriate assessment tools for the region.

The use of a clinical population, rigorous psychometric testing, and the inclusion of multiple correlates of aging expectations are notable strengths of this work. However, several limitations should be acknowledged. The study was based on a pilot sample that was relatively small and not randomly selected, which may limit the generalizability of the findings. Accordingly, the CFA results should also be interpreted as providing preliminary, pilot-level evidence for the factorial validity of the R-ERA-12. Nevertheless, participants were recruited from the Almaty Centre for AIDS Prevention and Control, which serves as the primary provider of HIV care for the entire city and rural areas, thereby ensuring a clinically relevant and diverse sample. Additionally, the cross-sectional design precludes any causal inferences. Future studies with larger and more representative samples are warranted to confirm these findings and to further examine the mechanisms shaping aging expectations among PLHIV in the region.

## Conclusion

The R-ERA-12 is a reliable and valid tool for assessing aging expectations, potentially among Russian-speaking PLHIV around the world. Participants reported particularly low expectations regarding physical health, with internalized HIV stigma emerging as a key determinant of negative perceptions. These findings underscore the need for interventions addressing stigma to promote more positive aging beliefs and overall well-being in this population.

## Data Availability

The datasets generated and/or analyzed during the current study are available from the corresponding author upon reasonable request.

## Footnotes

1
https://www.kobotoolbox.org/
